# Economic evaluation of health promotion interventions for older people: do applied economic studies meet the methodological challenges?

**DOI:** 10.1186/s12962-018-0100-4

**Published:** 2018-04-16

**Authors:** Kai Huter, Katarzyna Dubas-Jakóbczyk, Ewa Kocot, Katarzyna Kissimova-Skarbek, Heinz Rothgang

**Affiliations:** 1 SOCIUM Research Center on Inequality and Social Policy, University of Bremen, Mary-Somerville-Straße 5, 28359 Bremen, Germany; 2 High-profile Area of Health Sciences, University of Bremen, Bremen, Germany; 3 Health Economics and Social Security Department, Institute of Public Health, Jagiellonian University Medical College, Krakow, Poland

**Keywords:** Economic evaluation, Health promotion, Primary prevention, Older people

## Abstract

**Background:**

In the light of demographic developments health promotion interventions for older people are gaining importance. In addition to methodological challenges arising from the economic evaluation of health promotion interventions in general, there are specific methodological problems for the particular target group of older people. There are especially four main methodological challenges that are discussed in the literature. They concern measurement and valuation of informal caregiving, accounting for productivity costs, effects of unrelated cost in added life years and the inclusion of ‘beyond-health’ benefits. This paper focuses on the question whether and to what extent specific methodological requirements are actually met in applied health economic evaluations.

**Methods:**

Following a systematic review of pertinent health economic evaluations, the included studies are analysed on the basis of four assessment criteria that are derived from methodological debates on the economic evaluation of health promotion interventions in general and economic evaluations targeting older people in particular.

**Results:**

Of the 37 studies included in the systematic review, only very few include cost and outcome categories discussed as being of specific relevance to the assessment of health promotion interventions for older people. The few studies that consider these aspects use very heterogeneous methods, thus there is no common methodological standard.

**Conclusion:**

There is a strong need for the development of guidelines to achieve better comparability and to include cost categories and outcomes that are relevant for older people. Disregarding these methodological obstacles could implicitly lead to discrimination against the elderly in terms of health promotion and disease prevention and, hence, an age-based rationing of public health care.

**Electronic supplementary material:**

The online version of this article (10.1186/s12962-018-0100-4) contains supplementary material, which is available to authorized users.

## Main text

### Background

In the light of current demographic developments in most of the OECD countries health promotion interventions for older people are gaining importance. In the past, health promotion interventions have largely been focussed on children, young people and the working population. Now, the growing proportion of older people in the population of many OECD countries and the increase in their respective share of national health budget expenditure has amplified interest in health promotion interventions for older people. To justify the political implementation of these programmes there is a growing demand for health economic evaluations of health promotion interventions for older people, and the number of respective studies—though still not large—has increased considerably in recent years. These studies face several challenges. Alongside general methodological obstacles pertaining to the health economic evaluation of health promotion or public health interventions in general (e.g. [1]), there are obstacles to the health economic evaluation of health promotion interventions that specifically concern older people and that, if not considered appropriately, may disadvantage them. The general problems concern, for example, the attribution of effects, as it is more difficult to conduct randomized controlled trials against the background of the long time horizon of many of these interventions, as well as the fact that many of them are directed at populations or communities, while principles of evidence based medicine demand that relevant effects are proven for individuals [1, 2].

This paper focuses on the question to what extent specific requirements or methodological challenges of health economic evaluations of health promotion or preventive interventions *for older people* are taken into account in applied health economic evaluations. A summary of the main methodological challenges for the economic evaluation of health promotion interventions for older people that are discussed in the theoretical and methodological literature on economic evaluation will be presented in the following section. Following a more detailed analysis published in Huter et al. [3] four challenges emerge that are of specific relevance for the economic assessment of health promotion interventions for older people. These aspects may bias study results and thus severely affect the comparability of the results with results of health economic evaluations of interventions for other age groups or for different types of interventions. Accounting for them is crucial if studies are meant to serve as starting points for policy decisions concerning the allocation of resources for competing programmes.

To examine if and how these identified methodological requirements are taken into account in applied health economic studies, we draw on a systematic review on health economic evaluations of health promotion and primary prevention interventions for older people [4], which has been updated for this study and whose data is reanalysed with respect to the previously identified criteria. They are used to assess whether and how the methodological problems have been tackled in existing studies. The results of this analysis are then presented and discussed. As the results are very diverse they are described qualitatively. We close with a short appreciation of measures that would be needed to address the identified shortcomings of the existing research.

#### Main methodological challenges

The application of economic evaluation methods to health promotion interventions for older people encounters numerous methodological problems. These problems derive from three sources: (1) controversies related to economic evaluation methods themselves; (2) the distinctive design of health promotion programmes and (3) special features of older people as a target group.

General methodological problems pertaining to economic evaluations of health promotion interventions have been widely discussed [1, 5–9]: health promotion interventions in general tend to have a broader focus than curative interventions; their targets may not be limited to individual health gains but may also comprise social benefits or ‘beyond-health’ benefits for the individual and broader effects on the family, community or society as a whole. Health promotion activities may involve many different sectors, like education, housing, transportation, the environmental or social sector; many interventions rely on volunteer work or specific community involvement. The effects of many health promotion interventions are expected to unfold in a long-term perspective. For all these reasons a societal perspective is often recommended for health economic evaluations of health promotion, and sometimes generally required, as e.g. in the Netherlands and Sweden [6, 7, 10]. This implies the inclusion of a broad range of costs and benefits and builds the prerequisite of our argumentation.

An analysis of the literature on methodological problems of health economic evaluation targeting older people, health economic evaluation of health promotion and public health intervention in general, and specifically for older people shows that the following criteria are discussed as being most important and should be considered or at least reflected on for this specific target group [3]:

1. The appropriate measurement and valuation of informal caregiving (e.g. [11–13]);
2. The appropriate measurement and valuation of productivity costs (including unpaid work) (e.g. [14–16]);
3. Effects of the inclusion of costs unrelated to the intervention that incur in life years gained by an intervention (e.g. [17–19]);
4. The inclusion of ‘beyond-health’ benefits and/or the consideration of specific preferences of older people (in relation to social needs and health values) [20–24].

These four criteria thus serve as the analytical framework in this paper for assessing economic evaluation studies of health promotion activities for older people. They will be outlined in more detail in the following section. This review analyses whether and how they have been considered—or at least discussed—and what general statements can be made on the informative value of the included studies, especially with regard to comparability. We discuss why these criteria are important and why their omission in most of the assessed studies is problematic.

##### Informal caregiving

Informal care is relevant for the evaluation of health promotion for older people in a societal perspective in two respects. On the one hand, they may receive informal care. As it is often a defined target of health promotion for older people to avoid dependency on long-term care, informal care refers to *direct non*-*medical costs* that may be reduced by an intervention. This might be a reduction in informal caregiving time and/or effects on the health and well-being of the informal carer (e.g. [11, 13]). On the other hand, older people are also providers of informal care, and in this respect a valuation of costs of informal caregiver time could be considered as part of the *indirect or productivity costs* of an intervention (see next section).

##### Productivity costs

Productivity costs usually represent the economic productivity lost due to death and lost or an impaired ability to work or to engage in leisure activities due to morbidity [25]. Whether and to what extent these costs should be included in health economic evaluations is controversial in theory and practice [14]. Whether they are included at all depends ultimately on the normative framework of the evaluation. A welfarist perspective will include productivity costs; an extra-welfarist perspective will not weight health gains against productivity costs. A central argument relating to interventions for older people is that if productivity costs are included, costs for unpaid work have to be included as well; otherwise the societal value of seniors’ (mostly) unpaid work, e.g. volunteer work, household work and informal care, will be neglected. If the cost–effectiveness of interventions for older people is compared to cost–effectiveness results of interventions for younger people that include productivity costs, interventions for older people will be disadvantaged if the social value of their (unpaid) production is not considered [16].

##### Unrelated cost in added life years

While there is a consensus that future medical and non-medical costs that incur in life years gained by and related to an intervention should be included in the cost analysis, there is an on-going methodological debate over whether future costs that are unrelated to the intervention should be included (e.g. [17–19]). This refers to costs that occur as an indirect result of interventions that successfully prolong the life-span, for example higher costs for long-term care or health care costs that would not have occurred in a shorter life. With regard to the assessment of interventions for older people this is important, because when compared to interventions for younger people life-prolonging interventions for older people will be rated less cost–effective if these costs are included. The reason for this is that the present value of these costs will be higher due to their occurrence in the near future, while these costs will be valued lower for younger persons because future costs are discounted.

##### ‘Beyond-health’ benefits and specific preference structures of older people

As mentioned above, the goals of health promotion interventions are often not limited to individual health gains, but comprise ‘beyond-health’ benefits as well. Especially for older people, it is often difficult to distinguish between health needs and social needs, thus the social benefit of an intervention may often outweigh the health benefit. Integration into the community, inclusion, or increasing mobility are not always associated with an immediate improvement in health status, but they can be crucial outcomes of a health promotion programme. Social isolation, on the other hand, is negatively associated with health status and health-related quality of life in older people [26]. So while ‘beyond-health’ benefits are relevant for any target group, the crucial point here is that their importance increases for older people. Moreover, when valuing or comparing the effects of health promotion interventions it should be considered that older people (may) have different preferences than younger people [22, 27].

The identification of outcomes is not a primary task of health economic evaluations, but it is a specific requirement of health economic evaluations to condense the effects of an intervention to an outcome parameter that can be compared with costs. In the health economic evaluation of curative interventions by cost–effectiveness analysis (CEA) or cost–utility analysis (CUA) social outcomes are usually not considered. But in the assessment of health promotion—especially for older people—benefits may be underrated if such outcomes are not accounted for. It is especially a problem of the commonly used quality-adjusted life year (QALY) that it does not capture social benefits, and is especially inappropriate for covering the preference structures of older people, who for example regard physical functionalities as less important than younger people [20–22]. For these reasons it is important to assess whether and how social or general ‘beyond-health’ benefits are taken into account in health economic evaluations of health promotion interventions for older people if alternatives to the QALY are considered in cost–utility analyses, whether these problems are discussed at all—or at least mentioned.

### Methods

Methodological challenges of the economic evaluation of health promotion interventions for older people were identified by means of an analysis of a broad range of theoretical and methodological literature. Four issues were identified as most important with regard to the comparability and adequacy of economic studies. These four criteria will be applied as assessment criteria to analyse the studies included in a systematic review on the economic evaluation of health promotion and primary prevention interventions for older people [4].

#### Systematic review of empirica**l** studies

Within the original systematic review five electronic databases (MEDLINE via PubMed, EMBASE, The Cochrane Library, National Health Service Economic Evaluation Database, Health Technology Assessment Database via the Centre for Review and Dissemination) and the Internet websites of 23 institutions or projects related to the topic were searched for relevant articles. At a later stage, the reference lists of relevant papers were also screened. The database searches were conducted in July 2015; Internet websites of relevant institutions and projects were screened in the period August–September 2015 [4]. The review has been updated using the same data sources in March 2018.^1^ Search terms included various synonyms relating to older ‘population’ groups, health promotion OR primary prevention ‘intervention’ and economic evaluation as a ‘type of study’. The studies’ eligibility criteria included: (1) a target population of 65 years or older, (2) interventions classified as health promotion or primary prevention, (3) a full economic evaluation conducted. The studies included were those available in English, Polish or German. Publication years range from 2000 to 2018.

In terms of participants, studies focussing on the general population were only included if the outcomes were presented separately for any population group aged 65+. The interventions of interest were health promotion programmes whose scope was consistent with the WHO definition of health promotion [28] and/or one of the types of health promotion interventions specified by McKenzie et al. [29]. Primary prevention programmes were defined as those focused on precluding the initial occurrence of disease by risk reduction [30].

Two researchers assessed the eligibility of the studies independently of each other. Titles and abstracts were scrutinized to eliminate clearly irrelevant reports. The full text articles were obtained and appraised to ensure the compliance of the studies with the predetermined eligibility criteria. The extraction of data from the eligible studies was conducted by two researchers independently of each other. Besides a general quality assessment of the included studies on the basis of the ‘Drummond checklist for assessing economic evaluations’ [25], an additional assessment was performed by the same researchers based on the set of additional criteria related to the special character of the economic evaluation of health promotion programmes for older people for the studies of the systematic review and the studies that resulted in the update of this review. This assessment included the criteria outlined above (societal perspective, consideration of informal caregiving, productivity costs/unpaid work, costs related to life years gained, ‘beyond-health’ effects). This was double checked by a third (independent) researcher. The studies were examined to establish whether these criteria were included and how, or whether their exclusion is discussed as part of the studies’ limitations.

More information on the systematic review, including a detailed description of the studies under review and the general quality assessment, can be found in Dubas-Jakóbczyk et al. [4].

### Results

In addition to twenty-nine relevant studies that had been identified in the systematic review, eight studies have been identified in the update of the literature search, so thirty-seven studies are included in this assessment. The majority (n = 25) of the studies were economic evaluations of fall prevention strategies for older people [31–55]. Five studies focussed on problems of general or mobility disability [56–60], two on general health status [61, 62], two on lack of physical activity [63, 64], one on frailty [65], one on mental wellbeing [66] and a further one on oral health [67]. Regarding the economic evaluation methods used, 29 of the studies were CEAs, two included several natural outcome indicators [cost–consequence analyses (CCA)], and twelve included CUA as well. Five studies were CUAs alone and the remaining three studies were cost–benefit analyses (CBA), but only one of the CBAs included individual preferences by willingness-to-pay (WTP) rates for avoided mortality/morbidity.

The general quality of the studies was diverse, with 18 out of the 37 studies meeting the criteria for good quality as defined by Dubas-Jakóbcyk et al. [4] using the Drummond criteria. The most common problems were a lack of or imprecise information on particular elements of the economic evaluation process and the lacking inclusion of all relevant costs and consequences in relation to the respective perspective of the study. Additional file 1: Table S1 provides an overview of the main general characteristics of the studies included as well as a summary of the results of the assessment of the studies.

**Fig. 1:**
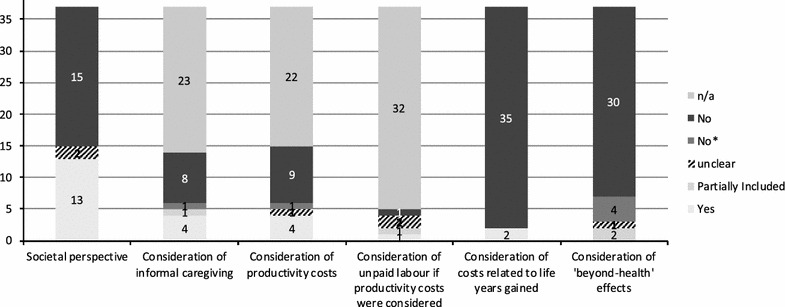
Frequency of the consideration of the assessed criteria in the included studies. *Not included, but mentioned as part of the study limitation or justification for exclusion provided (e.g. lack of data for calculation)

Figure 1 visualises the summarized result of the assessment of the studies with respect to special requirements for the economic evaluation of health promotion programmes for older people. It shows that these aspects were only considered in a very small number of studies. In general, the authors of the studies under review rarely included, discussed or commented on methodological problems resulting from the specific character of this type of evaluation. A *societal perspective* was adopted in only 13 of the 37 studies, and the tackling of the four specific challenges (see below) was attempted in less than half of the studies that claimed to take a societal perspective.

#### Informal care

*Costs of informal care* were taken into account in very few of the studies under review. Informal care was included as part of direct medical costs in only five of the 13 studies that claimed to take a societal perspective. One of these studies considered unpaid household work; the costs for this work were estimated by using nationally published estimates for unpaid household work [51]. A second study considered informal care, valued by the price of professional help as a replacement price [46]. The third study, a modelling study, used estimates on informal care costs provided by another study that valued informal caregiving time as lost leisure time (i.e. 3 €/h) [47, 68]. The fourth study valued informal care on the basis of shadow prices for unpaid work [45]; and a fifth study considered the cost of ‘hiring a babysitter’ and ‘lost income of any companion who accompanied the participant’ during the time of the intervention [57], which is a very limited approach to considering informal caregiving costs as part of the indirect cost of the intervention itself (programme costs). Of the remaining seven studies, only one mentioned the exclusion of informal care costs as a limitation [58]. One study was situated in a nursing home, thus informal care costs were not applicable here [52]. The others did not even discuss or mention the topic. The methods of measuring or estimating the time provided by informal caregivers differed from study to study insofar as they were reported. Effects on the informal caregivers’ health or well-being were not considered at all.

#### Productivity costs

Among the studies taking a societal perspective, four out of 13 included some sort of valuation of the participants’ time as part of the cost assessment, thus may have considered some kind of ‘*productivity costs*’. Three of these studies valuated only the time spent on the intervention as part of the implementation costs of the programme [47, 57, 63]. Only one study valuated the participants’ time spent in receipt of care, and thus included long-term effects of the intervention [46]; however, it did not provide any explanation or details as to how this was done. Apart from that, one single study mentioned the exclusion of older people’s time or production as a shortcoming of the study [62]. Two studies argued that productivity losses do not have to be assessed if the study population is predominantly retired [45, 60]. For one CBA it can be argued that these indirect costs were implicitly included as part of the valuation of WTP for averted morbidity and mortality [52].

The three studies that valuated the time spent on the intervention each used different methods. Only in one study were participants asked what they gave up to participate in the programme in question, so only this study provided a differentiated analysis with respect to paid work, volunteer work and informal care [63]. One study considered only the lost formal income of the participant or his/her companion or a possible baby-sitter’s wage [57]. One study valued the participants’ time as leisure time.

#### Unrelated costs in added years of life

Only two studies considered *costs for long*-*term care and health care in gained years.* One study included these costs in one version of its evaluations and it was shown that they had a substantial negative effect on the net benefit of the intervention; on the other hand, productivity or other social or individual benefits of time gained were not valued (though claiming a societal perspective) [62]. Still the authors concluded that in their (Swedish) policy context it did not seem reasonable from a public-decision making perspective to include this cost component. The other study developed a cost–effectiveness model to predict publicly funded health and aged care costs and QALYs over the remaining lifetime of frail older persons. While the model includes costs and QALY-benefits in gained years of life, the main concern of the study is the inclusion of long-term effects of interventions that are often disregarded due to the short observation periods. This study used the Markov model to predict publicly funded health and social care costs and QALYs to an age of 100 years maximum. The use of this model means that in the case of an intervention leading to life extension additional costs in gained years of life are included. Applied to the analysed physiotherapy-based intervention, effects on mortality were in fact negligible, so the added costs did not occur (however, the method used allows their inclusion) [65].

#### Outcomes: inclusion of ‘beyond-health effects’

Regarding the *inclusion of* ‘*beyond*-*health*’ *effects*, most studies included in the review used avoidance measures, like ‘falls prevented’, health status measures, or QALYs as outcome measures. When QALYs were used, however, no attempt was made to adjust for the preference structure of elderly which might differ significantly from the rest of the population. There were only two studies that included indicators of social benefits. The study by Iliffe et al. [63] included ‘self-efficacy for exercise’ and ‘social network size’ as additional indicators. The study by Mountain et al. [66] included ‘loneliness’, ‘general self-efficacy’ and ‘general well-being’ as secondary outcomes, in addition they assessed serious adverse events and whether they were related to the intervention. In the CBA by Wilson and Datta [52] social benefits may have been included to a limited extent by WTP for the avoidance of morbidity and mortality. Only four additional studies mentioned that there were social or intersectoral benefits that had not been considered [43, 44, 47, 62]. Johansson et al. [47], as one example, mentioned that cost–effectiveness may be underestimated because positive externalities of the programme were not considered. These may include a reduction of injuries in other age groups, due to the removal of street hazards, or increased social networks. Sahlen et al. [62] on the other hand merely mentioned that there may have been an ‘enjoyment’ factor compensating for the value of time used that had not been considered as well.

The limited comparability of results with interventions for other age groups, especially for studies that refer to QALYs as outcome indicators, was not mentioned in any of the studies.

### Discussion

In general, the analysis of the existing evidence on the economic evaluation of health promotion and primary prevention actions addressing older population groups showed huge differences in the methods applied as well as the overall quality of the studies [4]. The economic evaluations performed are very heterogeneous in relation to cost categories included and the presentation of outcomes. Therefore, the comparability of results is on the whole quite limited. Although the societal perspective is recommended for the economic evaluation of health promotion interventions, only 13 studies include this approach. Still, some of these studies do not differ from studies that claim to assume a patient/provider perspective with regard to costs and benefits included. These findings corroborate results discussed by Davis et al. [34] who argue that comparability of fall prevention studies is limited and seek to establish guidelines for conducting and reporting respective economic evaluations.

Heterogeneity is particularly obvious when it comes to the consideration of cost categories and outcomes that need special attention for health promotion interventions aiming at older people. This clearly indicates the need for a standard practice for conducting economic evaluations for health promotion interventions for older people. Theoretical debates on specific requirements of economic evaluations for this target group are rarely reflected on in economic evaluation practice so far.

#### Informal care

Research on the economic valuation of informal care has developed significantly over the last 10–15 years, but as yet this is not sufficiently reflected in applied health economic evaluations studies. Given the heterogeneity of methods used to include informal care, more research on the impact of different methods and an increasing consensus on how to value informal care, i.e. by the development of guidelines and/or reference cases, is necessary to attain a better comparability of results [11, 12, 69]. Given that it is often a defined target of health promotion interventions for older people to sustain self-sufficiency and avoid dependency on long-term care, costs for informal care should be included as part of the direct non-medical costs that may be avoided by an intervention. This comprises costs that are incurred to facilitate informal care and the valuation of the time spent by the uncompensated care-giver. Against the background of the missing consensus concerning the methods to assess informal care, and only a limited knowledge so far on the influences of the different methods on the cost–effectiveness-ratios, it is advisable to assess the impact of the inclusion by sensitivity analyses. If costs of informal care are not included in the study a sufficient explanation should be provided [12]. Effects on the health or well-being of the care-giver should be considered on the effect side of the economic evaluation.

If productivity costs are included (see below) informal care has to be considered as a value of ‘work-time’ lost or gained due to an intervention. This refers to indirect ‘programme costs’, i.e. value of time spent on the intervention itself as well as paid or unpaid ‘production loss’ or gain.

#### Productivity costs

The inclusion of productivity costs in health economic evaluations is controversial. As mentioned above, their inclusion is dependent on the normative framework of a study, and national health economic guidelines differ widely in their requirements and recommendations with regard to productivity costs in general and unpaid work in particular [70, 71]. A study by Krol and Brower on the general role of productivity costs related to unpaid work in current health economic evaluations confirms a general lack of awareness concerning these costs. This is reflected by a lack of clear guidance on how to measure and value lost unpaid work in major health economic textbooks and national health economic guidelines [70]. Concerning studies on elderly patient populations they conclude that it seems to be common reasoning “that productivity costs are irrelevant since patients are too old to be in paid profession” (ibid. 128).

Thus there are three reasons why the representation of productivity costs is unsatisfactory in health economic evaluations for older people. Firstly, the inclusion of productivity cost is contentious per se and not always required by national guidelines. Secondly, there is no consensus and a lack of guidance on how to measure and value productivity appropriately—in particular with regard to unpaid work. This is especially challenging if productivity costs are not only considered as part of the implementation costs of an intervention, but also comprise the long-term effects of an intervention. Thirdly, there is a disregard of productivity costs, because it is assumed that they are not relevant for this specific target group, which neglects the societal value of seniors’ unpaid work.

A discussion of the implications of different options of including productivity cost and especially unpaid work as part of productivity costs is beyond the scope of this paper. For future research it may be a starting point to report productivity costs separately from direct costs in order to allow a comparison of outcomes between studies. It may not be necessary—or due to lacking data feasible—to include productivity costs in every study; nevertheless, the non-inclusion of productivity costs should be justified very carefully. The omission of a differentiated analysis of productivity costs in the economic evaluation of interventions for older age groups will be problematic if study results are compared to those of interventions aiming at younger age groups that include productivity costs.

#### Unrelated costs in added years of life

The study in the review that considers costs for long-term care and health care in gained years in one version of its evaluations [62] shows that this has a significant effect on the net benefit of the intervention. These aspects and their possible discriminatory effects on interventions aiming at older people will need further attention in future research, as the inclusion or exclusion of unrelated future medical and long-term care costs may have notable distributional consequences [19], especially for interventions that increase the longevity of older people.

#### ‘Beyond-health’ benefits and specific preferences of older people

The assessment of the studies under review shows that beyond-health benefits are taken into account only to a very limited extent; and for the most part respective problems are not even mentioned. Limitations of the QALY to cover health benefits of older people or specific preferences of older people that may limit the comparability of the study results were not mentioned at all.

As it is methodologically challenging to include diverse effects in an economic evaluation compared to the assessment of a simple proxy indicator such as ‘number of falls prevented’, and social or beyond-health benefits are rarely included in health economic evaluations in general, it would be desirable that authors at least mention these problems, which is done in only very few studies.

One main reason for the omission of beyond-health effects is the methodical demand for economic studies to condense effects to a quantifiable outcome indicator. As yet, there is only scant methodological guidance on how to include social benefits in economic evaluations beyond cost–benefit analyses. Methodological guidelines were originally developed for clinical interventions, where social benefits are not usually taken into account. Most of the studies included in this review concern fall prevention—interventions with a clearly defined primary objective. As these are relatively similar to clinical interventions—compared to complex multi-layered health promotion or public health interventions—the necessity to include further-reaching effects may have appeared negligible. This might be true if the cost–effectiveness of fall prevention interventions is proven without considering social benefits, but the comparability of these results nevertheless remains limited. Thus, even when comparing results of different fall-prevention interventions it is beneficial to take into account in the cost–effectiveness assessment as to which interventions entail additional social benefits.

The QALY that comprises multidimensional health benefits is an approved outcome indicator in many national health economic guidelines. Its inclusion as an outcome indicator reflects the endeavour to achieve comparability with other interventions. Methodological debates on their limited capability to adequately capture quality-of-life gains for older people have only in recent years been reflected in attempts to develop more adequate outcome indicators. It is essential that concepts are developed that include beyond-health benefits and specific preferences of older people in health economic studies. One promising approach is the use of instruments such as ICECAP-O or ASCOT, which have been developed in recent years to measure the well-being of older people on the basis of their preferences (e.g. [24]). These instruments are recommended for use, for example, in economic evaluations of social care by the British National Institute for Health and Care Excellence [72]. Some authors recommend the use of cost consequence analysis in economic assessments in the field of health promotion, public health or other complex interventions [1, 73, 74]. Benefits such as, for example, general well-being, maintenance of independence, social inclusion, and safety are very relevant potential outcomes of health promotion interventions targeting older people; their ignorance might lead to an underestimation of their positive effects in economic evaluations.

Walter et al. [75] published a feasibility study in December 2017 on a home-based health promotion intervention for older people with mild frailty, that included an economic evaluation. Their conclusion is that a full randomized controlled trial including an economic evaluation of this intervention is feasible. The study included ICECAP-O and general well-being measures, considered informal care as part of the cost assessment, and while not explicitly referring to productivity costs, the time of the participants spent on caring responsibilities was at least reported. The study thus supports our claim for the inclusion of the categories discussed in this article in health economic evaluations and gives reason for hope for future studies with such a perspective.

### Conclusions

Overall, this assessment shows clearly that there is no established practice for conducting economic evaluations of health promotion interventions for older people from a societal perspective. Theoretical debates on specific requirements of the economic evaluation of interventions for older people have not permeated in the majority of applied health economic evaluations. Cost categories that are important for the economic evaluation of health promotion for older people are only partly reflected, and if they are considered, the methods used are very heterogeneous. Thus, there is a strong need for guidelines and reference cases to achieve better comparability. Social benefits are included only to a very limited extent; divergent preferences of older people are not considered at all. Instruments that have been developed in recent years to measure the well-being or specific preferences of older people are not yet used in existing studies. Their incorporation into future economic evaluations would be highly beneficial.

The authors of the existing studies only seldom analyse or comment on the limitations resulting from the direct application of economic evaluation methods typical for clinical interventions in the area of health promotion for older people.

We conclude:

- Methodological challenges are not sufficiently met in existing studies so far.
- A comparison of results of different economic evaluations, even of similar interventions, has to be carried out with great caution.
- A comparison of the cost–effectiveness results with other interventions or results for other age groups is not possible and therefore not advisable.

In order to deal with the specific problems of the economic evaluation of health promotion activities for the elderly, studies should address the four criteria described above. We recommend that these criteria should be included in the future quality assessment of health economic evaluations. Since no generally accepted practice exists as to how these criteria should be included, more research is necessary on the different approaches for their inclusion and on their respective effects on the outcomes. Disregarding these problems could implicitly lead to a discrimination of health promotion and disease prevention against older people and thus an age-based rationing of public health care. Health economic studies should at least be completely transparent regarding the methods they use to include these costs, or thoroughly justify why some cost categories may have been excluded.

## Additional file

**Additional file 1: Table S1.** Tabular summary of results. The table contains the results of the assessment of the four criteria specific for interventions targeting older people, some general characteristics and results of the general quality assessment of the studies included in the systematic review and its update.

## Abbreviations

CBA: cost–benefit analysis
CCA: cost–consequence analysis
CEA: cost–effectiveness analysis
CUA: cost–utility analysis
QALY: quality-adjusted life year
WTP: willingness-to-pay

## Footnotes

1 The National Health Service Economic Evaluation Database and the Health Technology Assessment Database via the Centre for Review and Dissemination have not been searched as they are no longer updated since 2015. The search in the databases and on websites identified 2.410 relevant citations. After removing duplicates 1.833 records were screened. Based on titles and abstracts 1.815 records were rejected. Based on full text analysis ten studies were excluded for not meeting the eligibility criteria. The most common reason for exclusion was the lack of separate outcomes for the population 65+ (six studies).
