# Supplementary material for: Economic evaluation of health promotion interventions for older people: do applied economic studies meet the methodological challenges?
Source: Cost Eff Resour Alloc. 2018 Apr 16;16:14. doi: 10.1186/s12962-018-0100-4 (PMC5902889; doi:10.1186/s12962-018-0100-4)
Supplement: Supplementary file 1 — Additional file 1: Table S1. Tabular summary of results. The table contains the results of the assessment of the four criteria specific for interventions targeting older people, some general characteristics and results of the general quality assessment of the studies included in the systematic review and its update. [file 12962_2018_100_MOESM1_ESM.pdf]

**Table S1. Tabular summary of results**		**Results of the assessment of the four criteria specific for interventions targeting older people**				**General characteristics of the studies, Results of the general quality assessment**			
No	Criteria / Study	Consideration of informal caregiving[3]	Consideration of productivity costs/ unpaid labour[4]	Consideration of costs related to the life years gained	Consideration of 'Beyond-health' effects	Societal perspective	Health Problem	Type of economic evaluation	General quality of the study[5]
	**Studies included in Dubas et al. 2017**								
1	Carande-Kulis et al. 2015 [31]	N/A	N/A	NO	NO	NO	Falls	CBA	moderate
2	Church et al. 2011 [32]	N/A	N/A	NO	NO	NO	Falls	CEA, CUA	moderate
3	Church et al. 2012 [33]	N/A	N/A	NO	NO	NO	Falls	CEA, CUA	poor
4	Davis et al. 2011 [34]	N/A	N/A	NO	NO	NO	Falls	CEA, CUA	good
5	Farag et al. 2015 [35]	N/A	N/A	NO	NO	NO	Falls	CUA	moderate
6	Frenkel et al. 2001 [67]	N/A	N/A	NO	NO	NO	Oral health	CCA	moderate
7	Frick et al. 2010 [36]	N/A	N/A	NO	NO	NO	Falls	CUA	moderate
8	Groessl et al. 2009 [56]	N/A	N/A	NO	NO	NO	General disability	CEA	moderate
9	Haas 2006 [37]	N/A	N/A	NO	NO	NO	Falls	CEA	moderate
10	Heinrich et al. 2013 [38]	N/A	N/A	NO	NO	NO	Falls	CEA	moderate
11	Hektoen et al. 2009 [39]	N/A	N/A	NO	NO	NO	Falls	CEA	good
12	Medical Advisory Secretariat 2008 [40]	N/A	N/A	NO	NO	NO	Falls	CEA	moderate
13	Irvine et al. 2010 [41]	N/A	N/A	NO	NO	NO	Falls	CEA	good
14	Kenkre et al. 2002 [42]	N/A	N/A	NO	NO	NO	Falls	CCA	poor
15	Munro et al. 2004 [61]	N/A	N/A	NO	NO	NO	General health status	CEA, CUA	moderate
16	Day et al. 2009 [43]	NO	NO	NO	NO[2]	UNCLEAR	Falls	CEA	moderate
17	Ling et al. 2008 [44]	NO	NO	NO	NO[2]	UNCLEAR	Falls	CBA	poor
18	Chen et al. 2008 [57]	(YES)[1]	YES/(YES)[1]	NO	NO	YES	General disability	CEA, CUA	poor
19	Hendriks et al. 2008 [45]	YES	NO	NO	NO	YES	Falls	CEA, CUA	good
20	Iliffe et al. 2014 [63]	NO	YES/YES	NO	(YES)[1]	YES	Lack of physical activity	CEA, CUA	good
21	Jenkyn et al. 2012 [46]	YES	YES/UNCLEAR	NO	NO	YES	Falls	CEA	good
22	Johansson et al. 2008 [47]	YES	YES/NO	NO	NO[2]	YES	Falls	CEA, CUA	good
23	Kronborg et al. 2006 [58]	NO[2]	NO	NO	NO	YES	General disability	CEA	good
24	Robertson et al. 2001a [48]	NO	NO	NO	NO	YES	Falls	CEA	moderate
25	Robertson et al. 2001b [49]	NO	NO	NO	NO	YES	Falls	CEA	good
26	Robertson et al. 2001c [50]	NO	NO	NO	NO	YES	Falls	CEA	moderate
27	Sahlen et al. 2008 [62]	NO	NO[2]	YES	NO[2]	YES	General health status	CUA	good
28	Salked et al. 2000 [51]	YES	NO	NO	NO	YES	Falls	CEA	moderate
29	Wilson & Datta 2001 [52]	N/A	UNCLEAR	NO	UNCLEAR	YES	Falls	CBA	good

**Table S1. Tabular summary of results**		**Results of the assessment of the four criteria specific for interventions targeting older people**				**General characteristics of the studies, Results of the general quality assessment**			
No	Criteria / Study	Consideration of informal caregiving[3]	Consideration of productivity costs/ unpaid labour[4]	Consideration of costs related to the life years gained	Consideration of 'Beyond-health' effects	Societal perspective	Health Problem	Type of economic evaluation	General quality of the study[5]
	**Studies included based on the update of the literature search**								
30	Cowper et al. 2017 [64]	N/A	N/A	NO	NO	NO	Lack of physical activity	CEA	good
31	Groessl et al. 2016 [59]	N/A	N/A	NO	NO	NO	Mobility disability	CEA/CUA	good
32	Karnon et al. 2017 [65]	N/A	N/A	YES	NO	NO	Frailty	CUA	good
33	McLean et al. 2015 [53]	N/A	N/A	NO	NO	NO	Falls	CEA, CUA	good
34	Mountain et al. 2017 [66]	N/A	N/A	NO	(YES)[1]	NO	Mental well-being	CEA, CUA	good
35	Mueller et al. 2015 [54]	N/A	N/A	NO	NO	NO	Fall-related fractures	CUA	good
36	Suijker et al. 2017 [60]	N/A[2]	N/A[2]	NO	NO	NO[2]	General disabilities	CEA, CUA	good
37	Isaranuwatchai et al. 2017 [55]	NO	NO	NO	NO	YES	Falls	CEA	moderate

Abbreviations: CEA: Cost effectiveness analysis; CUA: Cost utility analysis; CBA: Cost benefit analysis; CCA: Cost consequence analysis

[1] partially included

[2] mentioned as a part of the study limitation or justification for exclusion provided (e.g. lack of data for calculation)

[3] In the case of the studies with an applied perspective other than societal the criteria concerning informal caregiver time and productivity costs are in fact not applicable (N/A).

[4] If productivity costs, respectively a valuation of the participants' time is included, the second statement indicates whether unpaid labour is considered.

[5] Quality of the study as defined and assessed by Dubas-Jakóbczyk K, Kocot E, Kissimova-Skarbek K, Huter K, Rothgang H. Economic evaluation of health promotion and primary prevention actions for older people - a systematic review. Eur J Public Health. 2017; doi: 10.1093/eurpub/ckx030.
